# Clinical implementation of virtual reality in neurorehabilitation: a scoping review of barriers, facilitators, and motivational factors

**DOI:** 10.3389/fresc.2026.1912363

**Published:** 2026-09-07

**Authors:** Ahmed S. Alanazi, Mohammed M. Alrashidi, Reem Aljohani, Taif Aljohani, Hams Madkhali, Alhanouf Almutairi, Alaa M. Alqurafi, Saad Z. Alqahtani

**Affiliations:** Department of Physical Therapy, College of Medical Rehabilitation Sciences, Taibah University, Madinah, Saudi Arabia

**Keywords:** barriers, facilitators, implementation, neurorehabilitation, virtual reality

## Abstract

Virtual reality is increasingly used in neurorehabilitation because it provides interactive therapeutic experiences that can enhance patient engagement and support rehabilitation goals. Despite growing evidence supporting its potential benefits, the extent of its integration into routine clinical practice remains unclear. This scoping review aimed to examine the current clinical use of virtual reality in neurorehabilitation and identify barriers, facilitators, and motivational factors influencing its implementation.‏ Searches of seven electronic databases [PubMed, MEDLINE, Embase, Scopus, IEEE Xplore, the Physiotherapy Evidence Database (PEDro), and the Cochrane Central Register of Controlled Trials (CENTRAL)] were conducted to identify relevant studies published between 2010 and 2025. Eligible studies were screened according to predefined inclusion and exclusion criteria, and data were extracted and synthesised descriptively. Eleven studies met the inclusion criteria. Clinical use of virtual reality varied considerably across settings, with only a limited proportion of therapists reporting regular use. Stroke rehabilitation was the most common area of application. Increased patient engagement and positive patient feedback were identified as motivational factors, while improved infrastructure and therapist training were reported facilitators of implementation. Common barriers included limited technical support, insufficient time, inadequate training, and resistance to change. These findings indicate that although interest in virtual reality for neurorehabilitation is increasing, its adoption in clinical practice remains inconsistent and is influenced by organisational, technical, and human factors. Addressing these barriers may facilitate wider implementation and support the integration of virtual reality into routine neurorehabilitation services.

## 1 Introduction

Neurological conditions, including stroke, spinal cord injury, traumatic brain injury, Parkinson's disease, and multiple sclerosis, are among the leading causes of disability worldwide (1). Stroke is a major cause of acquired disability worldwide, with many survivors experiencing persistent upper-limb impairments that limit functional independence (2).

Achieving functional recovery in individuals with neurological conditions requires repetitive practice and augmented feedback to enhance motor learning (3, 4). Accordingly, the provision of safe, controlled, and task-oriented environments is essential to support effective rehabilitation (1).

In recent years, advances in rehabilitation technologies, particularly virtual reality (VR), have introduced new opportunities to address these needs. By enabling the simulation of interactive and goal-directed tasks, VR can enhance motor learning, promote engagement, and support task-specific training (5). These capabilities have contributed to its increasing integration as a complement to conventional rehabilitation approaches (3, 4, 6).

Virtual rehabilitation systems utilise hardware and software to create digital environments in which patients interact through body movements to achieve therapeutic goals (6). These systems often incorporate user-centred design principles, allowing interventions to be tailored to individual patient needs, abilities, and goals (7). Depending on the level of immersion, VR systems can be classified as fully immersive, semi-immersive, or non-immersive. In clinical rehabilitation, these systems may include head-mounted displays, screen-based applications, and motion-tracking technologies that support different therapeutic activities depending on therapeutic goals and clinical settings (6–8).

Key applications include VR-based exergames, which are designed to promote active participation through engaging and interactive tasks and facilitate the controlled practice of movement (8). In addition, VR systems enable the real-time capture of performance data, supporting continuous monitoring and adjustment of rehabilitation programmes (3).

Most previous reviews have focused on VR interventions and rehabilitation outcomes (8–10), while implementation-related factors were previously examined by Glegg and Levac (11) in 2018. However, more recent evidence on clinical implementation has since emerged, particularly regarding how VR is adopted and integrated within real-world neurorehabilitation settings.

This scoping review addresses this gap by focusing specifically on the clinical implementation of VR across neurorehabilitation settings. In this review, implementation refers to the clinical adoption and use of VR in neurorehabilitation practice, while also considering implementation-related factors, including feasibility, usability, barriers, facilitators, and motivational factors that influence its integration into routine clinical care. Therefore, there is a need to systematically examine how VR is currently used in neurorehabilitation practice, the conditions in which it is applied, and the factors influencing its implementation. Accordingly, the aim of this scoping review was to examine the current clinical use of VR in neurorehabilitation and identify key barriers, facilitators, and motivational factors influencing its implementation in practice.

## 2 Materials and methods

### 2.1 Study design

This scoping review followed the methodological framework proposed by Arksey and O’Malley (12) and further refined by Pham et al. (13). The framework comprises five stages: (1) identifying the research question; (2) identifying relevant studies; (3) selecting eligible studies; (4) charting the data; and (5) collating, summarising, and reporting the results. A scoping review methodology was selected because it is well suited to mapping heterogeneous evidence and examining emerging areas of research, such as the implementation of VR in neurorehabilitation practice. The review was conducted and reported in accordance with the Preferred Reporting Items for Systematic Reviews and Meta-Analyses Extension for Scoping Reviews (PRISMA-ScR). No review protocol was registered or published for this scoping review.

### 2.2 Guiding research questions

To develop a comprehensive understanding of the clinical use of VR in neurorehabilitation, this review was guided by the following questions:

1. How is VR currently used in clinical neurorehabilitation practice?
2. Which neurological conditions are addressed using VR in clinical neurorehabilitation settings?
3. What are the key barriers and facilitators influencing the adoption of VR in practice?
4. What motivational factors influence the use of VR in neurorehabilitation?

### 2.3 Identification of relevant studies

A comprehensive literature search was conducted across seven electronic databases: PubMed, MEDLINE, Embase, Scopus, IEEE Xplore, the Physiotherapy Evidence Database (PEDro), and the Cochrane Central Register of Controlled Trials (CENTRAL). The selected databases were considered relevant to the review question and the multidisciplinary nature of virtual reality in neurorehabilitation. The search included peer-reviewed studies published between 2010 and 2025. A combination of keywords, controlled vocabulary terms where applicable, and Boolean operators (AND/OR) was used to maximise the sensitivity and relevance of the search strategy. In addition, the reference lists of included studies were screened to identify any additional relevant studies. The detailed search strategy is provided in Supplementary Appendix S1.

### 2.4 Study selection process

Studies were eligible for inclusion if they:

1. Examined the use of VR in neurorehabilitation.
2. Involved participants with neurological conditions such as stroke, spinal cord injury, traumatic brain injury, Parkinson's disease, or multiple sclerosis.
3. Reported on clinical adoption, implementation, or factors influencing VR use in practice, rather than solely evaluating the clinical effectiveness of VR interventions.
4. Were published in English between 2010 and 2025.

For the purpose of this review, clinical application referred to the use of VR within routine neurorehabilitation practice or real-world rehabilitation settings, including studies evaluating its adoption, implementation, feasibility, usability, or integration into clinical care. Active videogame technologies were included only when they were used as rehabilitation tools within these settings and were evaluated in relation to clinical implementation rather than entertainment purposes.

Studies were excluded if they:

1. Focused on VR applications outside neurorehabilitation (e.g., orthopaedic rehabilitation or mental health).
2. Discussed technological advancements without clinical application.
3. Were case reports, editorials, opinion papers, or conference abstracts without available full text.

Search results were exported to an Excel spreadsheet for management and screening. Duplicates were identified and removed prior to screening. Three independent reviewers screened titles and abstracts against predefined inclusion criteria. The same three reviewers independently assessed the full texts for final inclusion. Any disagreements were resolved through discussion and consensus.

### 2.5 Data charting process

Data extraction was conducted using a predefined Excel template. The extracted data included:

- Author(s) and year of publication
- Study design
- Sample size and population characteristics
- VR technology type and modality
- Data collection methods
- Key findings
- Reported limitations

This process was conducted independently by three reviewers to minimise bias and ensure accuracy.

### 2.6 Synthesis of results

Findings from the included studies were synthesised descriptively to map the current clinical use of VR in neurorehabilitation. The synthesis focused on identifying patterns in clinical adoption, as well as key barriers, facilitators, and motivational factors influencing implementation. The results were grouped into thematic categories to provide a structured overview of the evidence and highlight areas requiring further investigation.

## 3 Results

### 3.1 Study selection

**Figure 1:**
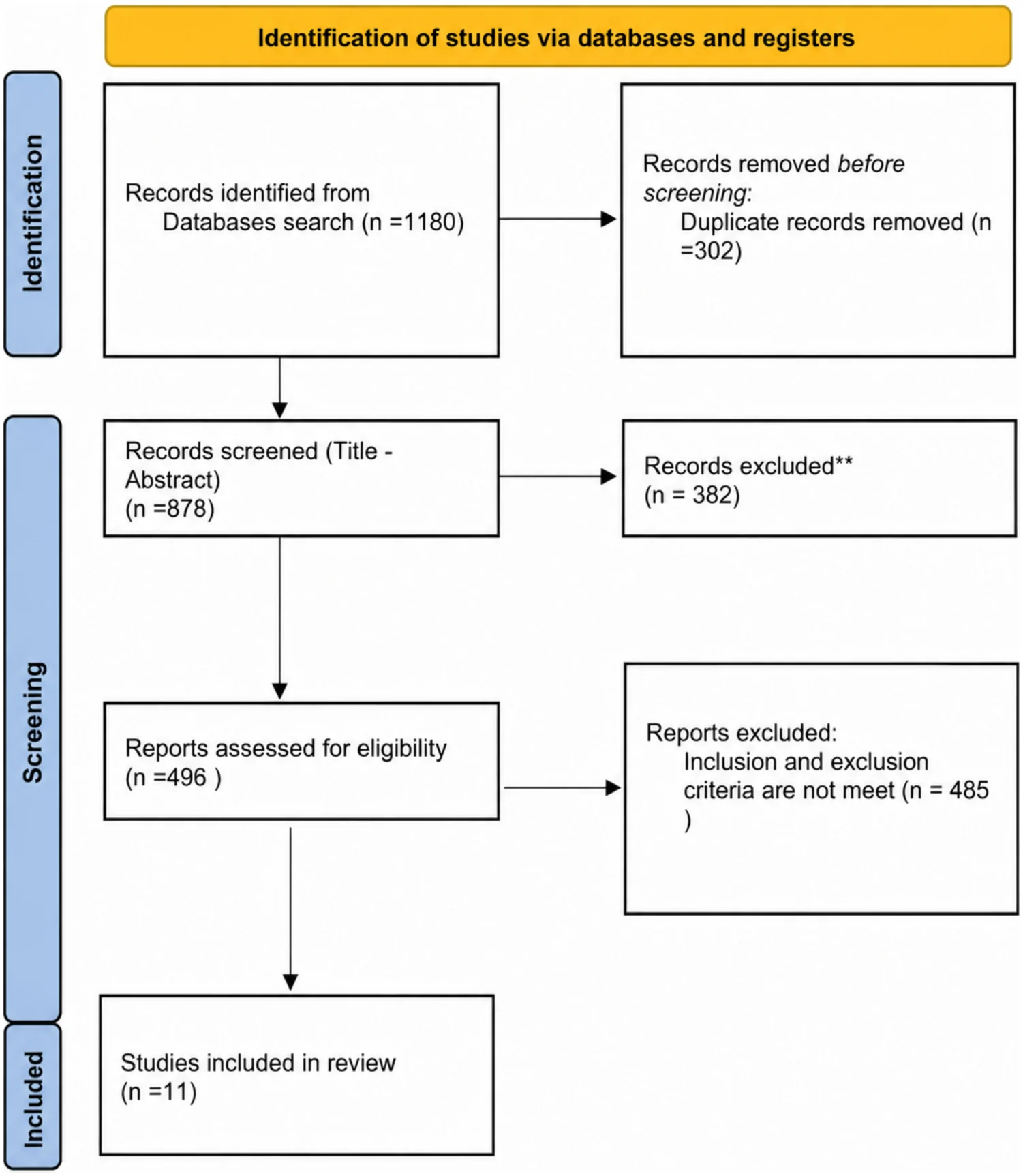
PRISMA flow diagram of the study selection process.

The database search identified 1,180 records from seven electronic databases [PubMed, MEDLINE, Embase, Scopus, IEEE Xplore, the Physiotherapy Evidence Database (PEDro), and the Cochrane Central Register of Controlled Trials (CENTRAL)]. After removing 302 duplicate records, 878 records remained for title and abstract screening. Of these, 496 records were assessed for full-text eligibility based on the predefined inclusion and exclusion criteria. Following full-text review, 11 studies met the eligibility criteria and were included in this scoping review. The main reasons for full-text exclusion were lack of clinical implementation focus, irrelevant population, and absence of implementation-related outcomes; however, exclusion reasons were not recorded quantitatively by category during full-text screening. The study selection process is presented in Figure 1.

### 3.2 Study characteristics

A total of 11 studies published between 2013 and 2025 were included in this review. The included studies comprised two cross-sectional surveys, three qualitative studies, and six studies employing other designs, including multi-site exploratory, descriptive quantitative feasibility, longitudinal observational, survey, multicentre, and quantitative designs. Sample sizes varied considerably, ranging from 8 healthcare professionals to 1,071 rehabilitation practitioners. The included studies investigated a range of VR technologies, with most examining multiple VR technologies and a smaller number focusing on non-immersive VR systems. Most studies explored the perspectives and experiences of physiotherapists, occupational therapists, and other rehabilitation professionals regarding the implementation of VR in clinical practice. Data collection methods included questionnaires, surveys, focus groups, semi-structured interviews, electronic medical record reviews, and standardized assessment tools. Several studies focused on specific neurological populations, including stroke, acquired brain injury, and paediatric neurological conditions.

Across the included studies, VR was generally perceived as a valuable adjunct to conventional rehabilitation, with reported benefits including improved patient motivation, engagement, balance, coordination, endurance, and functional performance. However, several barriers to implementation were consistently identified, including limited funding, inadequate training and technical support, lack of knowledge and confidence among clinicians, technological challenges, and organizational constraints. Facilitators commonly reported included positive clinician attitudes, patient motivation, availability of training opportunities, peer support, and institutional commitment to innovation. Detailed characteristics of the included studies are presented in Table 1.

**Table 1:** Characteristics of studies included in the scoping review.

Authors/year	Title	Type of study	Sample	VR technology	Data collection methods	Limitations	Key findings
Levac et al., 2017 (14)	Virtual Reality and Active Videogame-Based Practice, Learning Needs, and Preferences: A Cross-Canada Survey of Physical Therapists and Occupational Therapists	Cross-sectional survey	1,071 respondents (562 OTs, 506 PTs, and 3 dual-trained therapists)	Multiple VR modalities	Survey assessing demographics, VR/AVG experience, and learning needs	Variability in recruitment procedures and low response rate may have limited representativeness.	VR/AVG use in clinical practice was limited. Barriers included personal, social, technological, and organisational factors. Clinicians expressed a strong interest in further training.
Alrashidi et al., 2024 (4)	Virtual Reality Current Use, Facilitators and Barriers to Implementation in Paediatric Physiotherapy	Cross-sectional online survey	128 responses received (47 incomplete)	Multiple VR modalities	Survey including demographic information and the ADOPT-VR2 questionnaire	Small sample size, exclusion of non-APCP members, and short survey period.	VR use in paediatric physiotherapy was limited. Major barriers included insufficient funding and resources, while patient motivation, technology, and management support facilitated implementation.
Glegg et al., 2014 (15)	Using Virtual Reality in Clinical Practice: A Multi-Site Exploratory Study	Multi-site exploratory study	29 patients with acquired brain injury	Non-immersive	Clinical records and rehabilitation outcome measures collected across multiple rehabilitation centres	Technical issues and patient withdrawal affected intervention continuity.	The study reported improvements in balance, coordination, endurance, range of motion, and attention during rehabilitation.
Rathinam et al., 2025 (16)	Factors Influencing Virtual Reality Use in Paediatric Acquired Brain Injury Upper Limb Rehabilitation	Interpretative qualitative study	3 physiotherapists and 5 occupational therapists	Multiple VR modalities	Semi-structured interviews and focus groups	Limited participant numbers and technological infrastructure challenges.	Insufficient knowledge, training, and technological infrastructure were identified as major barriers to VR implementation.
Villada Castillo et al., 2024 (3)	Clinical Perceptions and Feasibility Analysis of a Virtual Reality Game for Post-Stroke Rehabilitation	Descriptive quantitative feasibility study	73 clinicians (23 general practitioners and 50 physiatrists)	Multiple VR modalities	Focus groups and customised surveys	High implementation costs, need for specialised training, and recruitment challenges.	VR was perceived as a feasible and acceptable adjunct to post-stroke rehabilitation and was considered motivating for patients.
Schmid et al., 2016 (17)	Therapists’ Perspective on Virtual Reality Training in Patients After Stroke	Qualitative study	9 therapists from three neurological rehabilitation centres	Non-immersive	Focus groups, audio-recorded discussions, and qualitative content analysis	Potential loss of meaning during translation of discussions.	Therapists perceived VR training as having a positive impact on rehabilitation outcomes.
Tatla et al., 2015 (18)	Therapists’ Perceptions of Social Media and Video Game Technologies in Upper Limb Rehabilitation	Qualitative study	10 therapists (4 OTs and 6 PTs)	Multiple VR modalities	Open-ended questionnaire	Small sample size and limited transferability of findings.	Therapists identified several barriers and facilitators influencing the adoption of gaming and social technologies in rehabilitation.
Pearce et al., 2024 (19)	Advanced Technology in a Real-World Rehabilitation Setting: Observational Study of Clinician Adoption and Implementation	Longitudinal observational study	80 clinicians and 269 patients	Multiple VR modalities	Electronic medical record review and business intelligence reports	Inconsistent outcome measurement and limited routine clinical data.	Adoption of advanced rehabilitation technologies varied across settings, with upper-limb robotic VR among the most frequently used technologies.
Hung et al., 2016 (20)	What Do Stroke Patients Look for in Game-Based Rehabilitation?	Survey study	30 stroke patients	Multiple VR modalities	Questionnaires and interviews using Likert-scale ratings	Limited methodological detail and absence of standardised outcome measures.	Patients preferred engaging game-based rehabilitation systems that incorporated familiar gaming features and interactive technologies.
Levac et al., 2016 (21)	A Knowledge Translation Intervention to Enhance Clinical Application of a Virtual Reality System in Stroke Rehabilitation	Multicentre study	6 PTs and 5 OTs	Non-immersive	ADOPT-VR questionnaire, confidence ratings, and system usability assessments	Small sample size during the sustainability phase.	Training interventions improved therapists’ confidence, knowledge, and skills related to VR implementation.
Glegg et al., 2013 (6)	Factors Influencing Therapists’ Adoption of Virtual Reality for Brain Injury Rehabilitation	Quantitative study	42 therapists	Non-immersive	ADOPT-VR survey and structured feedback questionnaires	Self-report measures and cross-sectional design may have introduced response bias.	Therapists reported positive attitudes towards VR. Lack of time and knowledge were common barriers, while training and peer support facilitated adoption.

### 3.3 Current clinical use of VR

Seven of the 11 included studies reported on the current clinical use of VR in neurorehabilitation. Overall, VR use in clinical practice was limited but demonstrated increasing adoption across a range of neurological conditions, including stroke, spinal cord injury, traumatic brain injury, and acquired brain injury. In the cross-Canada survey by Levac et al. (14), which included 1,071 rehabilitation practitioners, 46% reported having used VR or active video games (AVG) in clinical practice at least once, while 12% reported regular use. Similarly, limited implementation was reported in paediatric physiotherapy settings in the United Kingdom (4).

In individuals with acquired brain injury, VR was used to target balance, coordination, endurance, range of motion, and attention. However, discontinuation was reported in 59% of patients, with reasons including competing priorities, technical difficulties, or lack of suitability (6). VR interventions were commonly delivered in short sessions at moderate frequencies (two to five sessions per week) and were typically introduced approximately five weeks after admission (6).

In stroke rehabilitation, therapists reported positive perceptions of VR, particularly regarding patient engagement and motivation (17). VR was commonly used as a complementary intervention, especially when rehabilitation activities were personalised to individual patient needs (3). Use was more frequently reported in outpatient and community settings than in inpatient settings, particularly for individuals with stroke and spinal cord injury. Upper-limb robotic VR systems were reported more frequently than immersive VR systems (19). Commercial gaming platforms, including Kinect and Wii, were commonly used because of their compatibility, accessibility, and ease of use (14, 18).

### 3.4 Motivational factors

#### 3.4.1 Patient-related motivational factors

Five of the 11 included studies reported motivational factors influencing the implementation of VR in clinical rehabilitation. The Young Persons’ Advisory Group (YPAG) identified several strategies to encourage the use of VR in rehabilitation, highlighting its appeal to young patients (16). Among individuals undergoing stroke rehabilitation, audio-visual effects and the novelty of VR-based rehabilitation were identified as important motivating features (21). Other factors related to motivation and engagement included positive rewards and feedback, leveraging children's interest in video games, enhanced control of body movements, favourable cost-benefit perceptions, and realistic virtual environments, all of which contributed to sustained patient engagement in rehabilitation programmes (16).

#### 3.4.2 Clinician-related motivational factors

Healthcare professionals also reported several factors that encouraged the implementation of VR in clinical practice. Prior experience with VR among healthcare professionals was identified as an important motivating factor (16). A survey found that 91.8% of healthcare professionals recognised the advantages of VR-based rehabilitation systems such as Motion Health VR and reported positive perceptions of VR as a therapeutic tool (3). Therapists also reported that VR-based gaming and social interaction features could provide a psychologically safe environment for patients whose disabilities might otherwise limit social participation (18). In addition, therapists identified perceived benefits to patients (40%) and the motivational value of VR (29%) as primary reasons for intending to increase future use of VR in clinical practice (4).

### 3.5 Facilitators of implementation

#### 3.5.1 Organisational and technology-related facilitators

Several facilitators influencing VR implementation were identified across the included studies. Access to evidence supporting VR benefits, sufficient time for implementation, and the availability of technical and managerial support were reported as factors supporting successful adoption (9). Infrastructure improvements, including reliable internet access, user-friendly software, and well-maintained hardware, were also reported as important factors supporting implementation (19). Additional facilitators included the potential cost savings associated with reduced travel and accommodation requirements for patients living in rural areas, as well as improved treatment adherence and accountability (18).

#### 3.5.2 Clinician-related facilitators

Peer influence was identified as a facilitator, with therapists more likely to adopt VR when it aligned with existing treatment approaches (19). Ongoing collaboration between clinicians and technology developers was highlighted as a mechanism for improving the accessibility and usability of VR applications in rehabilitation settings (4, 6). Collaboration between healthcare professionals and engineers was also reported as supporting the development and implementation of VR systems in stroke rehabilitation (17).

### 3.6 Barriers to implementation

#### 3.6.1 Organisational barriers

Several barriers to VR implementation were reported across the included studies. Limited funding was identified as a barrier, affecting both the acquisition and maintenance of VR equipment (4). A lack of trained personnel, limited access to technical support, time constraints within rehabilitation services, and insufficient training opportunities for therapists were also reported as barriers (4).

#### 3.6.2 Technology-related barriers

Technical and logistical challenges included unreliable internet connections, complex system interactions, limited physical space for VR use, limited accountability within gaming systems, and difficulties translating virtual performance into real-world functional improvements (6, 19) Additional concerns included inconsistencies in outcome measurement and limited lengths of patient stay (19, 20). Privacy and confidentiality concerns were also identified as factors influencing implementation (19, 22).

#### 3.6.3 Patient-related barriers

In paediatric rehabilitation settings, parental concerns regarding screen time and technology use were identified as additional barriers (16). Cognitive impairments, agitation, difficulties understanding virtual environments, and concerns regarding age appropriateness were also reported as factors that could reduce patient engagement with VR interventions (16).

#### 3.6.4 Clinician-related barriers

Resistance to change among healthcare professionals, a preference for conventional rehabilitation approaches, limited education, and restricted access to technology were reported as significant barriers to VR adoption (18).

## 4 Discussion

The aim of this scoping review was to examine the clinical implementation of VR in neurorehabilitation. The findings highlight a growing yet inconsistently implemented field. Although several included studies reported perceived benefits associated with VR use, such as improved patient engagement and support for rehabilitation activities, the primary focus of this review was the implementation of VR rather than its clinical effectiveness. The findings indicate that VR adoption remains variable and is influenced by multiple patient-, clinician-, technology-, and organisational-level factors.

The included studies highlighted several practical challenges related to the design and applicability of current VR systems. Therapists reported that many VR applications are insufficiently aligned with activities of daily living (ADLs) and do not adequately reflect patient-centred rehabilitation goals (3, 17, 18). This disconnect underscores the need for more task-oriented VR designs that better replicate real-world functional activities. In this context, interprofessional collaboration between VR developers and rehabilitation professionals was consistently emphasised as a critical factor in bridging the gap between technological capabilities and clinical requirements (3, 17, 18).

Clinical decision-making regarding the use of VR was also influenced by patient-related and organisational factors. Therapists reported that treatment intensity and duration were often adapted according to patient endurance, clinical priorities, and time constraints within rehabilitation settings (6). Shorter, moderate-intensity VR programmes were generally perceived as more feasible, particularly in inpatient environments (6). In addition, VR was frequently used selectively during specific phases of rehabilitation and was sometimes discontinued when barriers such as technical issues, competing treatment goals, or reduced patient motivation were encountered (6). Therapist attitudes also emerged as an important determinant of VR implementation. While most clinicians expressed positive perceptions regarding the potential of VR to support rehabilitation activities and increase patient engagement, concerns were raised regarding its compatibility with hands-on therapeutic approaches and the additional time required for setup and integration into routine workflows (3, 14).

Several studies also explored broader implementation factors, including knowledge, training, and organisational readiness (23). Key themes included improving awareness of VR technologies, investing in clinician training, strengthening infrastructure, and supporting implementation strategies (23). These elements highlight the importance of a systematic and multilevel approach to integrating VR into clinical practice (23). Additionally, therapists emphasised the need to balance VR-based interventions with real-world physical activities, as improvements observed in virtual environments may not fully translate into functional performance in daily life (3). These findings can be understood within a multilevel implementation perspective, where barriers and facilitators operate across different levels. Technology-related factors, including usability, system design, and compatibility with rehabilitation goals, reflect intervention-level determinants, whereas training, infrastructure, funding, and organisational readiness represent organisational and health-system determinants. This distinction highlights that successful VR implementation requires not only improvements in technology design but also supportive clinical environments, appropriate resources, and structured implementation strategies.

Positive perceptions of VR were further supported by findings from surveys and qualitative studies, which indicated that many clinicians are familiar with game-based rehabilitation systems and have experimented with commercially available technologies such as Wii and Kinect (14, 18). Although VR was generally viewed as a promising therapeutic tool, there remains a need for more user-friendly, adaptable, and clinically relevant systems to support widespread adoption (20).

Although the included studies investigated a range of VR technologies, meaningful comparisons between fully immersive, semi-immersive, and non-immersive systems were not possible. Most studies evaluated multiple VR modalities or did not report implementation outcomes separately for each modality. Consequently, the influence of VR modality on implementation, usability, motivation, feasibility, safety, cost, equipment requirements, or organisational requirements remains an important evidence gap that should be addressed in future research.

The role of motivation and engagement was also highlighted, with gaming elements and interactive features identified as potential facilitators of patient participation. Although motivation, engagement, usability, acceptability, and satisfaction are closely related, they represent distinct implementation constructs and should not be interpreted interchangeably. Motivation reflects the willingness of patients or clinicians to use VR, whereas engagement relates to the extent of active participation during rehabilitation. Usability concerns the ease with which VR systems can be used in clinical practice, while acceptability reflects users’ perceptions of the appropriateness of the technology within rehabilitation settings. Variations in patient demographics and preferences, as well as concerns related to technology use, influenced the extent to which these factors supported implementation. Furthermore, existing evidence on game-based rehabilitation suggests that while benefits may be present, they are often modest and dependent on factors such as patient condition and intervention design (18).

From an implementation perspective, several facilitators were identified, including patient motivation, clinician acceptance, and organisational support (11, 24). Institutional factors such as resource allocation, availability of equipment, and management support played a critical role in enabling VR adoption (10). Observational learning and exposure to successful VR implementation among peers also contributed to increased confidence and willingness among clinicians to adopt the technology. In addition, broader system-level initiatives and funding programmes were recognised as important drivers for facilitating the integration of VR into clinical practice (4, 11, 24). Despite these facilitators, multiple barriers continue to limit the widespread adoption of VR in neurorehabilitation. Commonly reported challenges included limited resources, insufficient technical support, lack of time, and inadequate training opportunities (11). Some of these barriers may reflect broader challenges associated with implementing digital health technologies rather than barriers specific to VR. These barriers were particularly evident in high-demand clinical environments, where therapists prioritised established treatment approaches over newer technologies (4). These findings are consistent with previous literature indicating that, despite growing interest, the translation of VR into routine practice remains constrained by practical and organisational limitations (14).

Addressing these barriers will require targeted strategies at multiple levels. Enhancing clinician training through structured educational programmes, improving system usability, and ensuring adequate technical support are essential steps (25). Financial considerations must also be addressed through appropriate funding mechanisms to support the acquisition and maintenance of VR systems (15, 26). Additionally, efforts to increase awareness, demonstrate clinical value, and integrate VR into existing rehabilitation pathways may help reduce resistance to change and support wider adoption (4, 9, 15, 17).

This review has several limitations that should be acknowledged. First, the number of included studies was relatively small, which may limit the generalisability of the findings. Second, only English-language studies were included, which may have resulted in the exclusion of relevant research. Third, as a scoping review, no formal critical appraisal of study quality was conducted; therefore, the strength of the evidence may vary across studies. In addition, heterogeneity in VR technologies, implementation-related constructs, study designs, participant characteristics, and outcome measures limited direct comparison across studies. Therefore, the findings should be interpreted as a thematic and descriptive synthesis rather than an estimate of the prevalence or relative importance of implementation determinants. Publication bias cannot be excluded, and the limited number of implementation-focused studies should be considered when interpreting the findings. Finally, the included studies provided limited information regarding adverse events associated with VR use, such as cybersickness, dizziness, nausea, visual discomfort, or fatigue, as well as accessibility considerations for individuals with cognitive, sensory, visual, communication, or age-related impairments. Consequently, these aspects could not be comprehensively synthesised in the present review.

Despite these limitations, the findings highlight important directions for future research. Greater standardisation in study design, reporting, and outcome measures is needed to support comparison across studies and strengthen the evidence base. Future research should prioritise large-scale longitudinal studies and implementation-focused investigations to examine the long-term feasibility, usability, acceptability, sustainability, and integration of VR into clinical practice. Furthermore, exploring strategies to overcome implementation barriers and enhance clinician engagement will be essential to support the successful integration of VR into neurorehabilitation.

## 5 Conclusion

This scoping review provided an overview of the current evidence regarding the clinical implementation of VR in neurorehabilitation. Although VR is perceived as a promising adjunct to conventional rehabilitation and may support rehabilitation participation and patient engagement, the current evidence base remains limited and heterogeneous, preventing firm conclusions regarding its clinical effectiveness or widespread implementation. The findings indicate that VR adoption is influenced by multiple factors, including organisational support, technical requirements, clinician training, and patient-related determinants. Key barriers, such as resource limitations, insufficient training, and technical challenges, continue to affect implementation, whereas clinician acceptance, patient motivation, and organisational readiness may facilitate integration into clinical practice. Given the limited and heterogeneous evidence base, particularly regarding actual clinical adoption, future research should focus on standardised implementation frameworks, robust study designs, and longitudinal investigations to prospectively evaluate implementation outcomes, feasibility, sustainability, and long-term integration of VR within routine neurorehabilitation settings.

## References

[1] Khan F Amatya B Galea MP Gonzenbach R Kesselring J. Neurorehabilitation: applied neuroplasticity. J Neurol. (2017) 264(3):603–15. 10.1007/s00415-016-8307-9 27778158

[2] Belagaje SR. Stroke rehabilitation. Continuum (Minneap Minn). (2017) 23(1, Cerebrovascular Disease):238–53. 10.1212/CON.0000000000000423 28157752

[3] Villada Castillo JF López JF Muñoz JE Gallo OH. Clinical perceptions and feasibility analysis of a virtual reality game for post-stroke rehabilitation. TecnoLógicas. (2024) 27(61). 10.22430/22565337.3180

[4] Alrashidi M Tomlinson RJ Buckingham G Williams CA. Virtual reality current use, facilitators and barriers to implementation in paediatric physiotherapy: cross-sectional online survey of UK paediatric physiotherapists. Disabil Rehabil Assist Technol. (2025) 20(3):585–91. 10.1080/17483107.2024.2393695 39180393

[5] Dong Y Liu X Tang M Huo H Chen D Wu Z. A haptic-feedback virtual reality system to improve the box and block test (BBT) for upper extremity motor function assessment. Virtual Real. (2023) 27(2):1199–219. 10.1007/s10055-022-00727-2

[6] Glegg SMN Holsti L Velikonja D Ansley B Brum C Sartor D. Factors influencing therapists’ adoption of virtual reality for brain injury rehabilitation. Cyberpsychol Behav Soc Netw. (2013) 16(5):385–401. 10.1089/cyber.2013.1506 23713844

[7] Alrashidi MM Evans JO Tomlinson RJ Williams CA Buckingham G. Usability of a virtual reality circle drawing task to assess upper-limb motor performance in children and young people with cerebral palsy: pilot study. BMC Pediatr. (2026) 26(1):244. 10.1186/s12887-026-06626-8 41735958 PMC13037249

[8] Kim W-S Cho S Ku J Kim Y Lee K Hwang H-J. Clinical application of virtual reality for upper limb motor rehabilitation in stroke: review of technologies and clinical evidence. J Clin Med. (2020) 9(10):3369. 10.3390/jcm9103369 33096678 PMC7590210

[9] Voinescu A Sui J Fraser DS. Virtual reality in neurorehabilitation: an umbrella review of meta-analyses. J Clin Med. (2021) 10(7):1478. 10.3390/jcm10071478 33918365 PMC8038192

[10] Massetti T da Silva TD Crocetta TB Guarnieri R de Freitas BL Bianchi Lopes P. The clinical utility of virtual reality in neurorehabilitation: a systematic review. J Cent Nerv Syst Dis. (2018) 10:1179573518813541. 10.1177/1179573518813541 30515028 PMC6262495

[11] Glegg SMN Levac DE. Barriers, facilitators and interventions to support virtual reality implementation in rehabilitation: a scoping review. PM&R. (2018) 10(11):1237–51.e1. 10.1016/j.pmrj.2018.07.004 PMC6752033 30503231

[12] Arksey H O'Malley L. Scoping studies: towards a methodological framework. Int J Soc Res Methodol. (2005) 8(1):19–32. 10.1080/1364557032000119616

[13] Pham MT Rajić A Greig JD Sargeant JM Papadopoulos A McEwen SA. A scoping review of scoping reviews: advancing the approach and enhancing the consistency. Res Synth Methods. (2014) 5(4):371–85. 10.1002/jrsm.1123 26052958 PMC4491356

[14] Levac D Glegg S Colquhoun H Miller P Noubary F. Virtual reality and active videogame-based practice, learning needs, and preferences: a cross-Canada survey of physical therapists and occupational therapists. Games Health J. (2017) 6(4):217–28. 10.1089/g4h.2016.0089 28816511

[15] Glegg SM Holsti L Stanton S Hanna S Velikonja D Ansley B. Using virtual reality in clinical practice: a multi-site exploratory study. NeuroRehabilitation. (2014) 35(3):563–77. 10.3233/NRE-141152 25238866

[16] Rathinam C Farr W Ray D Gupta R. Factors influencing virtual reality use in paediatric acquired brain injury upper limb rehabilitation: a qualitative study. BMJ Open. (2025) 15(1):e083120. 10.1136/bmjopen-2023-083120 39819954 PMC11751983

[17] Schmid L Glässel A Schuster-Amft C. Therapists’ perspective on virtual reality training in patients after stroke: a qualitative study reporting focus group results from three hospitals. Stroke Res Treat. (2016) 2016(1):6210508. 10.1155/2016/6210508 28058130 PMC5183768

[18] Tatla SK Shirzad N Lohse KR Virji-Babul N Hoens AM Holsti L. Therapists’ perceptions of social media and video game technologies in upper limb rehabilitation. JMIR Serious Games. (2015) 3(1):e2. 10.2196/games.3401 25759148 PMC4373832

[19] Pearce LMN Pryor J Redhead J Sherrington C Hassett L. Advanced technology in a real-world rehabilitation setting: longitudinal observational study on clinician adoption and implementation. J Med Internet Res. (2024) 26:e60374. 10.2196/60374 39753210 PMC11729780

[20] Hung Y-X Huang P-C Chen K-T Chu W-C. What do stroke patients look for in game-based rehabilitation: a survey study. Medicine (Baltimore). (2016) 95(11):e3032. 10.1097/MD.0000000000003032 26986120 PMC4839901

[21] Levac D Glegg SMN Sveistrup H Colquhoun H Miller PA Finestone H. A knowledge translation intervention to enhance clinical application of a virtual reality system in stroke rehabilitation. BMC Health Serv Res. (2016) 16(1):557. 10.1186/s12913-016-1807-6 27716179 PMC5052802

[22] Cain A Gunby T Winstein C Demers M. Advancing stroke rehabilitation: the role of wearable technology according to research experts. Disabil Rehabil Assist Technol. (2025) 20(5):1460–9. 10.1080/17483107.2025.2459326 39874139 PMC12187557

[23] Brassel S Power E Campbell A Brunner M Togher L. Recommendations for the design and implementation of virtual reality for acquired brain injury rehabilitation: systematic review. J Med Internet Res. (2021) 23(7):e26344. 10.2196/26344 34328434 PMC8367177

[24] Alt Murphy M Pradhan S Levin MF Hancock NJ. Uptake of technology for neurorehabilitation in clinical practice: a scoping review. Phys Ther. (2024) 104(2):pzad140. 10.1093/ptj/pzad140 37856528 PMC10851848

[25] Patel SB Iqbal FM Lam K Acharya A Ashrafian H Darzi A. Characterizing behaviors that influence the implementation of digital-based interventions in health care: systematic review. J Med Internet Res. (2025) 27:e56711. 10.2196/56711 40505130 PMC12203025

[26] Rashid A Mukhtar T Najam S Khalid R Rasheed H Qamar M. Advancing neurorehabilitation through virtual reality and robotics: a critical narrative review of motor recovery technologies. J Health Wellness Community Res. (2025) e601. 10.61919/rgff5r13

