# Supplementary material for: Clinical implementation of virtual reality in neurorehabilitation: a scoping review of barriers, facilitators, and motivational factors
Source: Front Rehabil Sci. 2026 Sep 7;7:1912363. doi: 10.3389/fresc.2026.1912363 (PMC13590962; doi:10.3389/fresc.2026.1912363)
Supplement: Supplementary file 1 [file Supplementaryfile1.docx]

Supplementary Appendix S1

Detailed Search Strategy

A comprehensive literature search was conducted across seven electronic databases: PubMed, MEDLINE, Embase, Scopus, IEEE Xplore, the Physiotherapy Evidence Database (PEDro), and the Cochrane Central Register of Controlled Trials (CENTRAL). The search strategy was adapted to the requirements of each database while maintaining the same conceptual framework. Searches combined terms related to virtual reality, neurological conditions, implementation, barriers and facilitators, and rehabilitation. Searches were limited to peer-reviewed studies published in English between 2010 and 2025. The final database searches were conducted in December 2025.

### 1. PubMed

("Virtual Reality"[Mesh] OR "virtual reality"[Title/Abstract] OR "virtual reality therapy"[Title/Abstract] OR "active video games"[Title/Abstract] OR gaming[Title/Abstract])

AND

("Stroke"[Mesh] OR "Brain Injuries, Traumatic"[Mesh] OR "Spinal Cord Injuries"[Mesh] OR "Parkinson Disease"[Mesh] OR "Multiple Sclerosis"[Mesh] OR "Cerebral Palsy"[Mesh] OR neurorehabilitation[Title/Abstract] OR "neurological rehabilitation"[Title/Abstract] OR stroke[Title/Abstract] OR "traumatic brain injury"[Title/Abstract] OR "brain injury"[Title/Abstract] OR "spinal cord injury"[Title/Abstract] OR "Parkinson's disease"[Title/Abstract] OR "multiple sclerosis"[Title/Abstract] OR "cerebral palsy"[Title/Abstract])

AND

(implementation[Title/Abstract] OR "clinical practice"[Title/Abstract] OR "technology adoption"[Title/Abstract] OR "knowledge translation"[Title/Abstract] OR "implementation science"[Title/Abstract])

AND

(barriers[Title/Abstract] OR facilitators[Title/Abstract] OR "motivational factors"[Title/Abstract] OR motivation[Title/Abstract] OR enablers[Title/Abstract] OR acceptance[Title/Abstract])

AND

("Physical Therapy Modalities"[Mesh] OR "Occupational Therapy"[Mesh] OR "Rehabilitation"[Mesh] OR "physical therapy"[Title/Abstract] OR physiotherapy[Title/Abstract] OR "occupational therapy"[Title/Abstract] OR rehabilitation[Title/Abstract])

**Limits:** English language; publication years 2010–2025.
**Final search:** December 2025.

### 2. MEDLINE

("Virtual Reality" OR "virtual reality therapy" OR "active video games" OR gaming)

AND

("Stroke" OR "Brain Injuries, Traumatic" OR "Spinal Cord Injuries" OR "Parkinson Disease" OR "Multiple Sclerosis" OR "Cerebral Palsy" OR neurorehabilitation OR "neurological rehabilitation" OR stroke OR "traumatic brain injury" OR "brain injury" OR "spinal cord injury" OR "Parkinson's disease" OR "multiple sclerosis" OR "cerebral palsy")

AND

(implementation OR "clinical practice" OR "technology adoption" OR "knowledge translation" OR "implementation science")

AND

(barriers OR facilitators OR "motivational factors" OR motivation OR enablers OR acceptance)

AND

("Physical Therapy" OR physiotherapy OR "Occupational Therapy" OR rehabilitation)

**Limits:** English language; publication years 2010–2025.
**Final search:** December 2025.

### 3. Embase

('virtual reality'/exp OR 'virtual reality',ab OR 'virtual reality therapy',ab OR 'active video games',ab OR gaming,ab)

AND

('stroke'/exp OR 'traumatic brain injury'/exp OR 'spinal cord injury'/exp OR 'Parkinson disease'/exp OR 'multiple sclerosis'/exp OR 'cerebral palsy'/exp OR neurorehabilitation,ab OR 'neurological rehabilitation',ab OR stroke,ab OR 'traumatic brain injury',ab OR 'brain injury',ab OR 'spinal cord injury',ab OR 'Parkinson disease',ab OR 'multiple sclerosis',ab OR 'cerebral palsy',ab)

AND

(implementation,ab OR 'clinical practice',ab OR 'technology adoption',ab OR 'knowledge translation',ab OR 'implementation science',ab)

AND

(barrier*,ab OR facilitator*,ab OR 'motivational factors',ab OR motivation,ab OR enabler*,ab OR acceptance,ab)

AND

('physical therapy'/exp OR 'occupational therapy'/exp OR 'rehabilitation'/exp OR 'physical therapy',ab OR physiotherapy,ab OR 'occupational therapy',ab OR rehabilitation,ab)

**Limits:** English language; publication years 2010–2025.
**Final search:** December 2025.

### 4. Scopus

TITLE-ABS-KEY ("virtual reality" OR "virtual reality therapy" OR "active video games" OR gaming)

AND TITLE-ABS-KEY ("neurorehabilitation" OR "neurological rehabilitation" OR stroke OR "traumatic brain injury" OR "brain injury" OR "spinal cord injury" OR "Parkinson's disease" OR "multiple sclerosis" OR "cerebral palsy")

AND TITLE-ABS-KEY (implementation OR "clinical practice" OR "technology adoption" OR "knowledge translation" OR "implementation science")

AND TITLE-ABS-KEY (barrier* OR facilitator* OR "motivational factors" OR motivation OR enabler* OR acceptance)

AND TITLE-ABS-KEY ("physical therapy" OR physiotherapy OR "occupational therapy" OR rehabilitation)

**Limits:** English language; publication years 2010–2025.
**Final search:** December 2025.

### 5. IEEE Xplore

("virtual reality" OR "virtual reality therapy" OR "active video games" OR gaming)

AND

("neurorehabilitation" OR "neurological rehabilitation" OR stroke OR "traumatic brain injury" OR "brain injury" OR "spinal cord injury" OR "Parkinson's disease" OR "multiple sclerosis" OR "cerebral palsy")

AND

(implementation OR "clinical practice" OR "technology adoption" OR "knowledge translation" OR "implementation science")

AND

(barriers OR facilitators OR "motivational factors" OR motivation OR enablers OR acceptance)

AND

("physical therapy" OR physiotherapy OR "occupational therapy" OR rehabilitation)

**Limits:** English language; publication years 2010–2025.
**Final search:** December 2025.

### 6. Physiotherapy Evidence Database (PEDro)

Because PEDro uses a more restricted search interface, simplified searches based on the main concepts were conducted using the Advanced Search function:

"virtual reality" AND neurorehabilitation

"virtual reality" AND stroke

"virtual reality" AND neurological rehabilitation

"virtual reality" AND brain injury

"virtual reality" AND spinal cord injury

"virtual reality" AND Parkinson

"virtual reality" AND multiple sclerosis

"virtual reality" AND cerebral palsy

"active video games" AND rehabilitation

Records retrieved through these searches were subsequently screened for clinical implementation, adoption, feasibility, usability, barriers, facilitators, and motivational factors.

**Limits:** Publication years 2010–2025.
**Final search:** December 2025.

### 7. Cochrane Central Register of Controlled Trials (CENTRAL)

("virtual reality" OR "virtual reality therapy" OR "active video games" OR gaming)

AND

("neurorehabilitation" OR "neurological rehabilitation" OR stroke OR "traumatic brain injury" OR "brain injury" OR "spinal cord injury" OR "Parkinson's disease" OR "multiple sclerosis" OR "cerebral palsy")

AND

(implementation OR "clinical practice" OR "technology adoption" OR "knowledge translation" OR "implementation science")

AND

(barriers OR facilitators OR "motivational factors" OR motivation OR enablers OR acceptance)

AND

("physical therapy" OR physiotherapy OR "occupational therapy" OR rehabilitation)

**Limits:** English language; publication years 2010–2025.
**Final search:** December 2025.

Reference lists of all included studies were also screened to identify additional potentially eligible studies. Search results were exported for duplicate removal, title and abstract screening, full-text assessment, and final study selection according to the predefined inclusion and exclusion criteria.
